# Supplementary material for: Bisphenol A (BPA) Directly Activates the G Protein-Coupled Estrogen Receptor 1 and Triggers the Metabolic Disruption in the Gonadal Tissue of Apostichopus japonicus
Source: Biology (Basel). 2023 May 31;12(6):798. doi: 10.3390/biology12060798 (PMC10295501; doi:10.3390/biology12060798)
Supplement: Supplementary file 1 [file biology-12-00798-s001.zip › Figures S2-S17 - Original lmages for Blots.pdf]

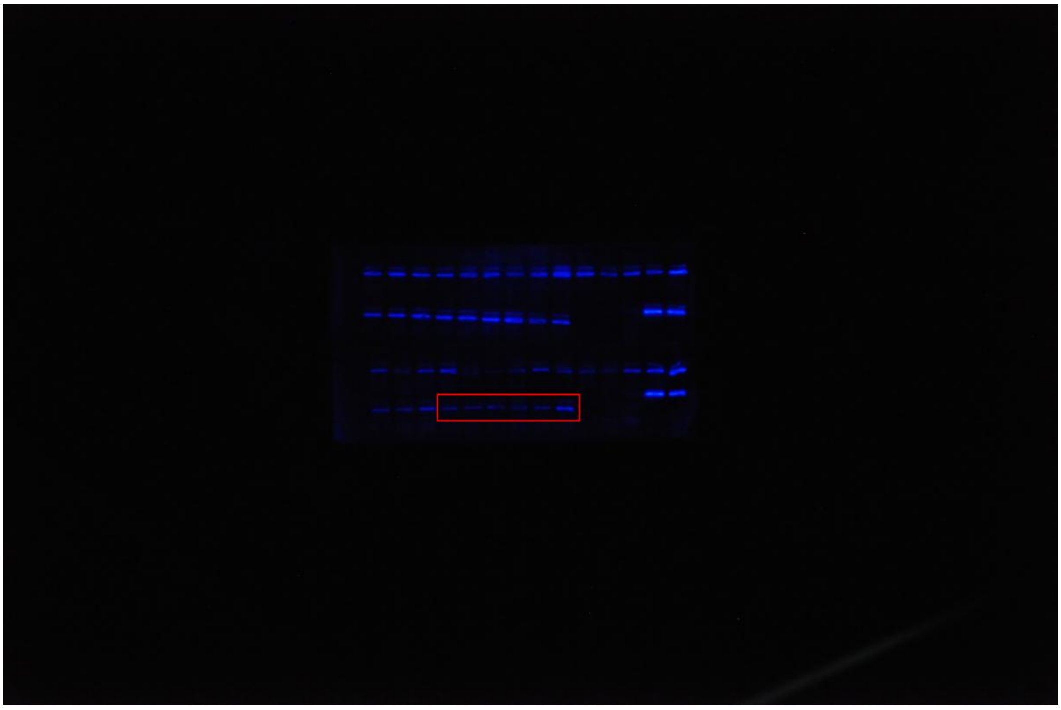

Figure S2. In the red box, from left to right, the phosphorylated ERK1/2 of blank (untransfected) cells, control (transfected with empty vector pEGFP-N1) cells, and AjGPER1-EGFP expressing cells treated with DMSO or E_2 (100 nM) for 10 min, respectively. Corresponding to Figure 5A in the manuscript.

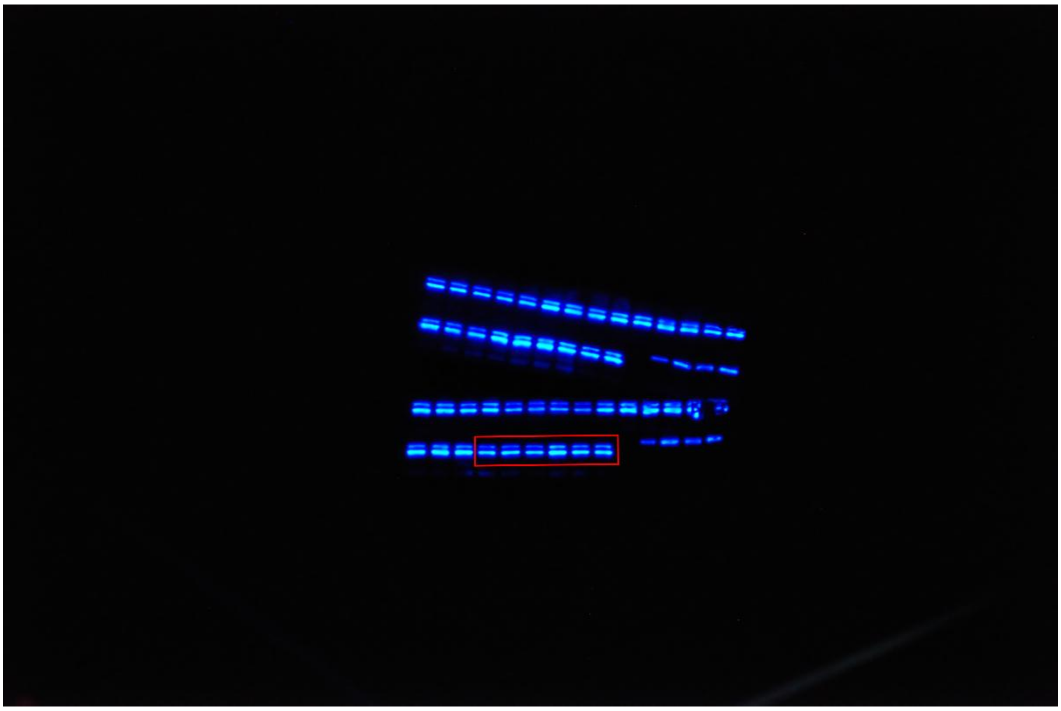

Figure S3. In the red box, from left to right, the total ERK1/2 of blank (untransfected) cells, control (transfected with empty vector pEGFP-N1) cells, and AjGPER1-EGFP expressing cells treated with DMSO or E_2 (100 nM) for 10 min, respectively. Corresponding to Figure 5A in the manuscript.

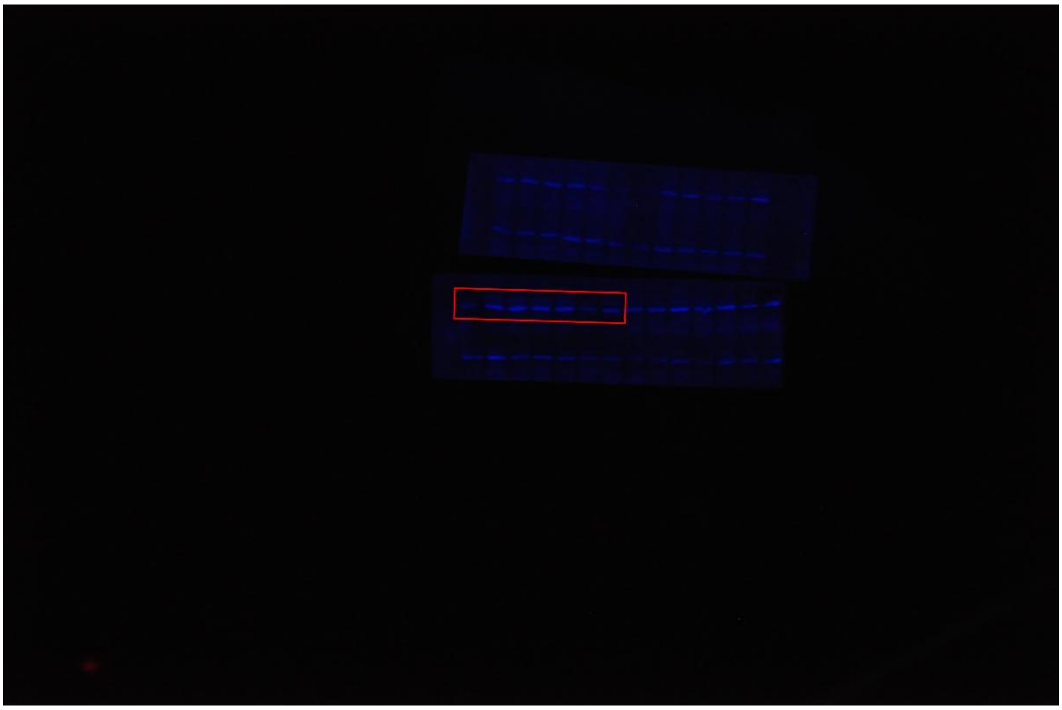

Figure S4. In the red box, from left to right, time course of E_2 (100 nM) treatment induced ERK1/2 phosphorylation in AjGPER1-EGFP-expressing cells. Corresponding to Figure 5B in the manuscript.

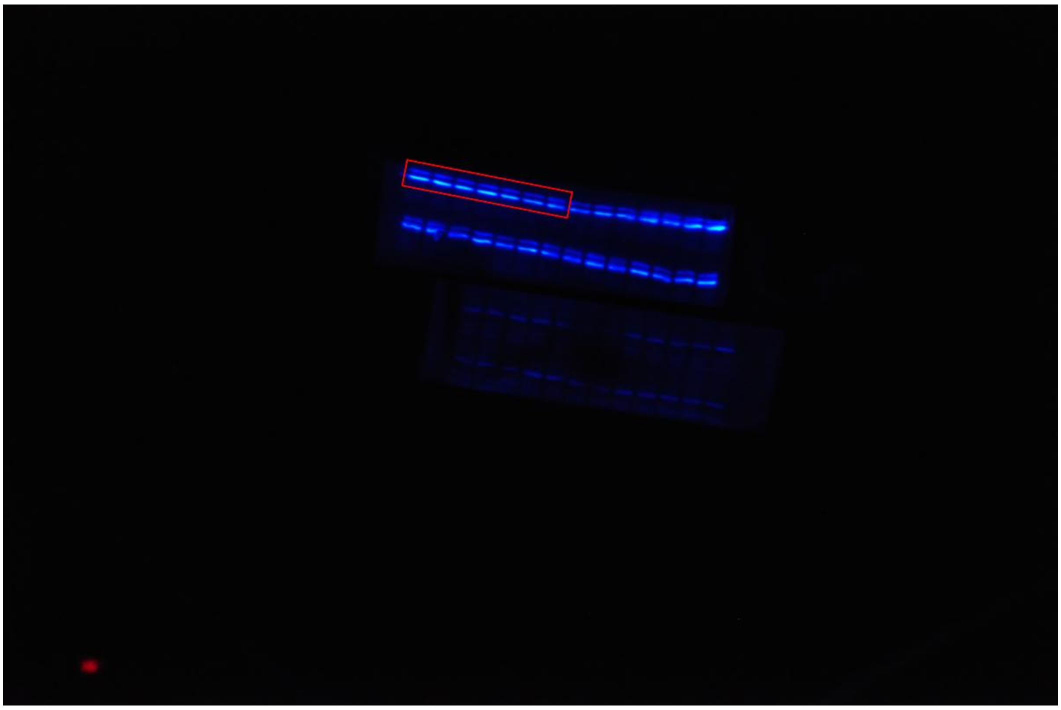

Figure S5. In the red box, from left to right, time course of E_2 (100 nM) treatment total ERK1/2 in AjGPER1-EGFP-expressing cells. Corresponding to Figure 5B in the manuscript.

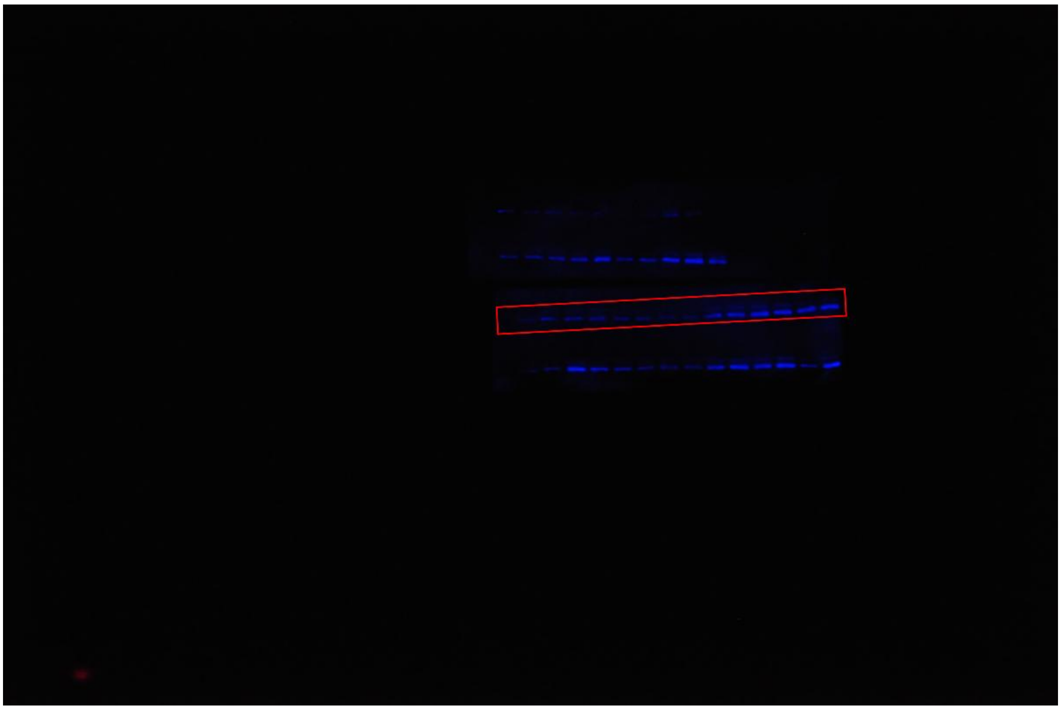

Figure S6. In the red box, from left to right, the phosphorylated ERK1/2 of Serum-starved AjGPER1-expressing cells were pretreated with PKA inhibitor (H89, 10 μM), or DMSO, for 1 h before E_2 (100 nM) administration. Corresponding to Figure 6A and B in the manuscript.

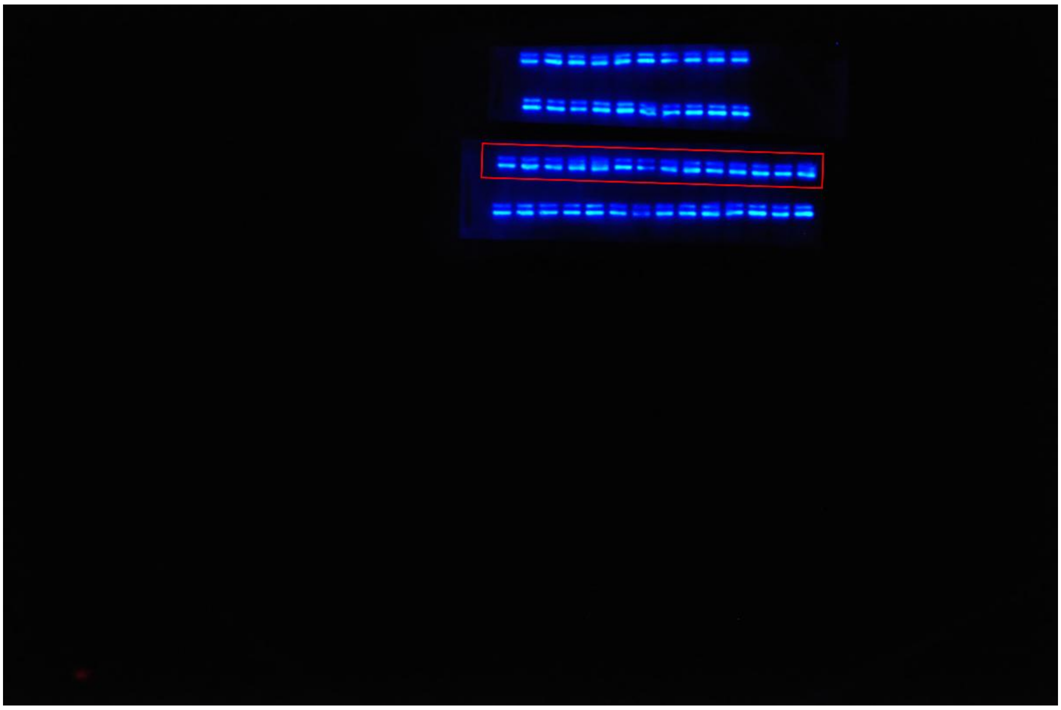

Figure S7. In the red box, from left to right, the total ERK1/2 of Serum-starved AjGPER1-expressing cells were pretreated with PKA inhibitor (H89, 10 μM), or DMSO, for 1 h before E_2 (100 nM) administration. Corresponding to Figure 6 A and B in the manuscript.

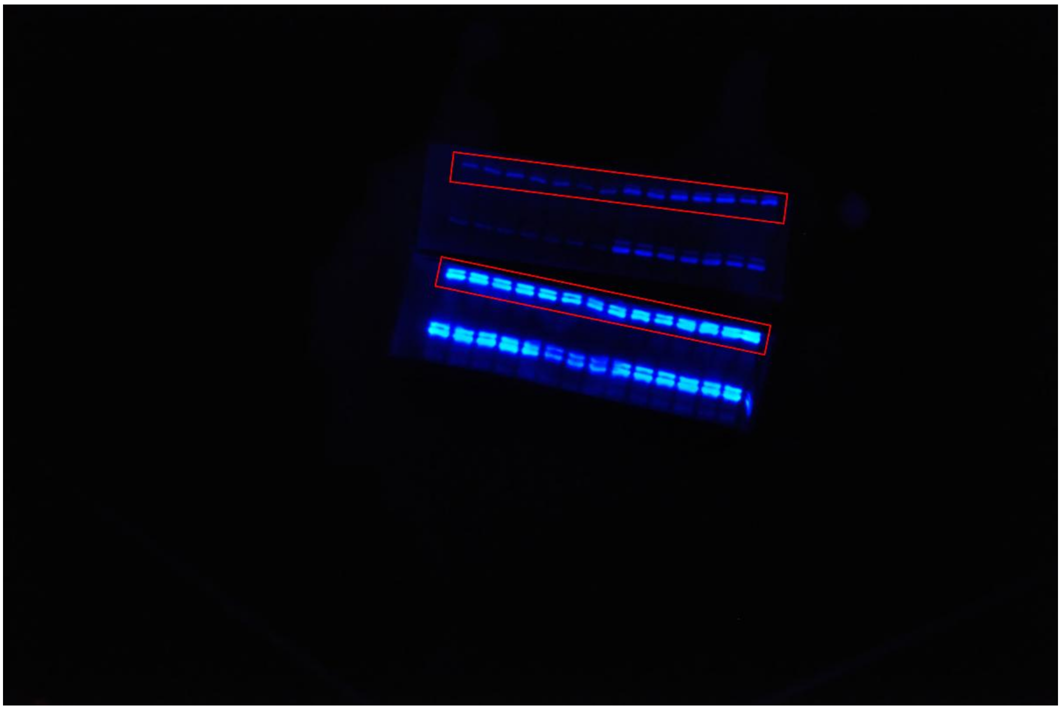

Figure S8. In the red box, from top to bottom, from left to right, the phosphorylated ERK1/2 and total ERK1/2 of Serum-starved AjGPER1-expressing cells were pretreated with PKC inhibitor (Go 6983, 1 μM), or DMSO, for 1 h before E_2 (100 nM) administration. Corresponding to Figure 6C in the manuscript.

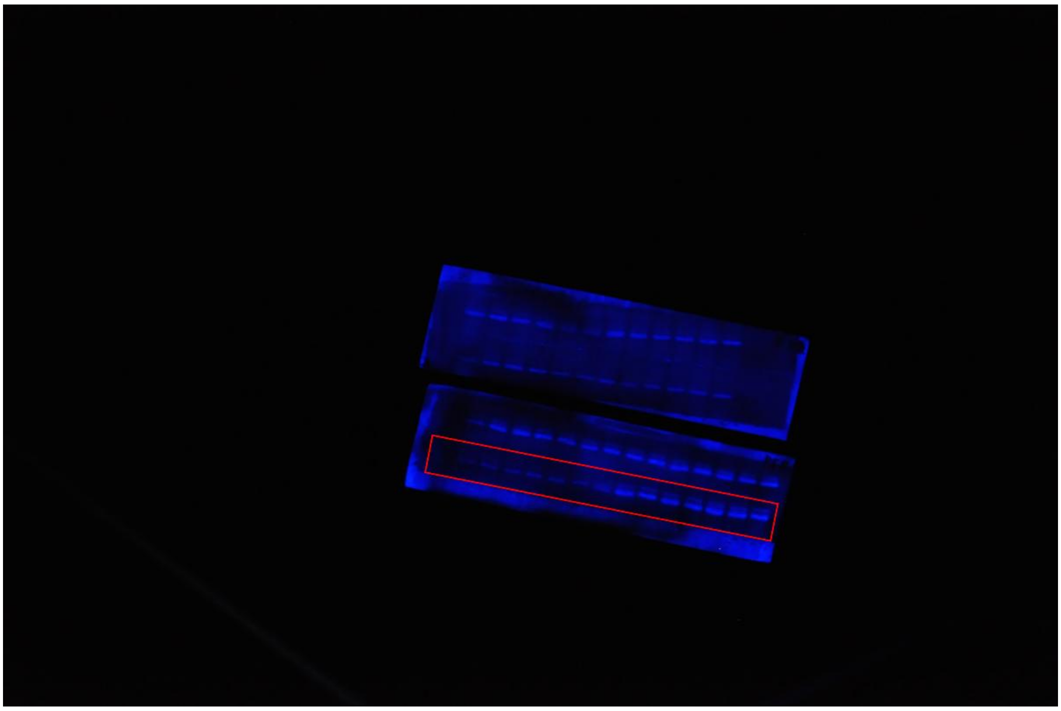

Figure S9. In the red box, from left to right, the phosphorylated ERK1/2 of Serum-starved AjGPER1-expressing cells were pretreated with $G_{\alpha q}$ protein inhibitor (FR900359, 1 μM), or DMSO, for 1 h before E_2 (100 nM) administration. Corresponding to Figure 6D in the manuscript.

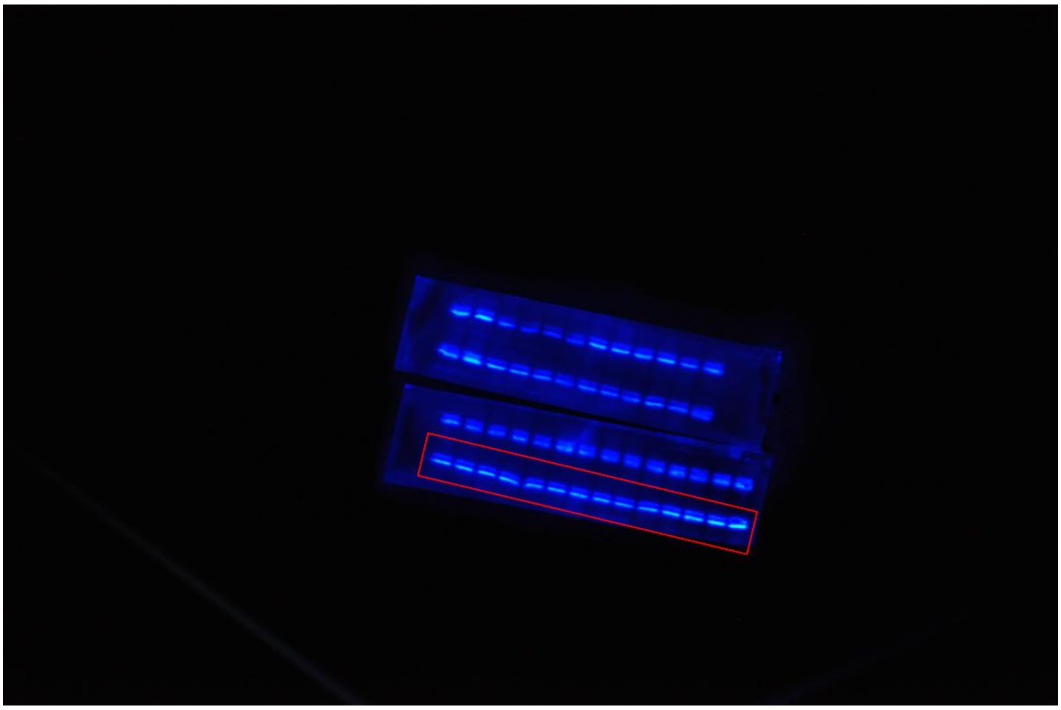

Figure S10. In the red box, from left to right, the total ERK1/2 of Serum-starved AjGPER1-expressing cells were pretreated with $G_{\alpha q}$ protein inhibitor (FR900359, 1 μM), or DMSO, for 1 h before E_2 (100 nM) administration. Corresponding to Figure 6D in the manuscript.

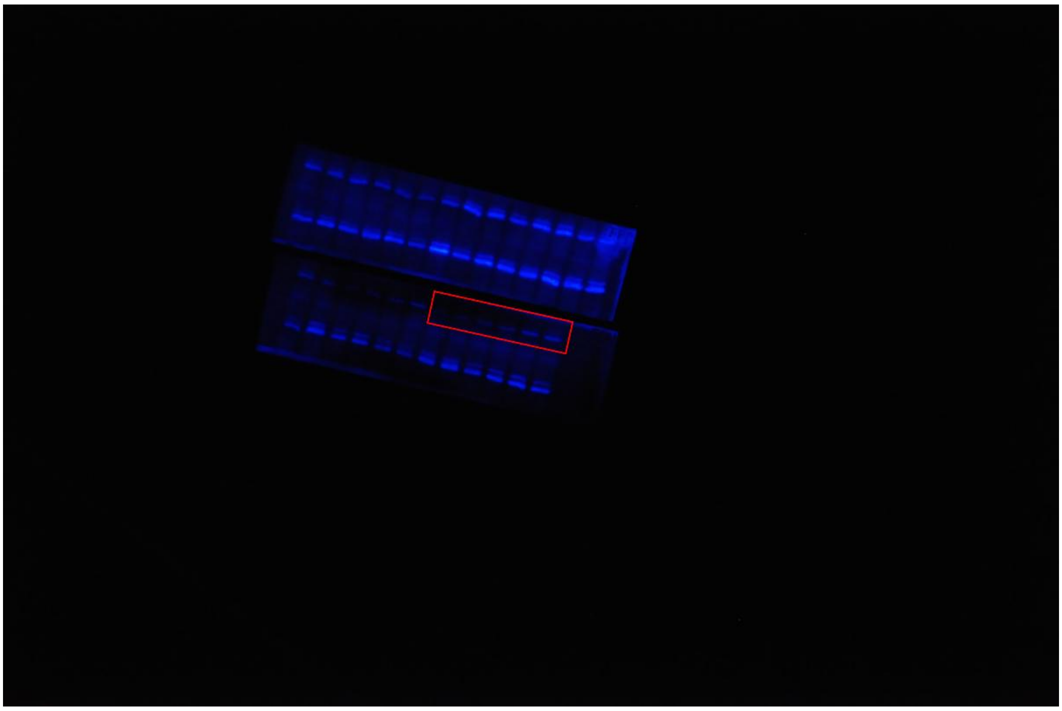

Figure S11. In the red box, from left to right, the phosphorylated ERK1/2 of blank (untransfected) cells, control (transfected with empty vector pEGFP-N1) cells, and AjGPER1-EGFP expressing cells treated with DMSO or BPA (100 nM) for 10 min, respectively. Corresponding to Figure 7A in the manuscript.

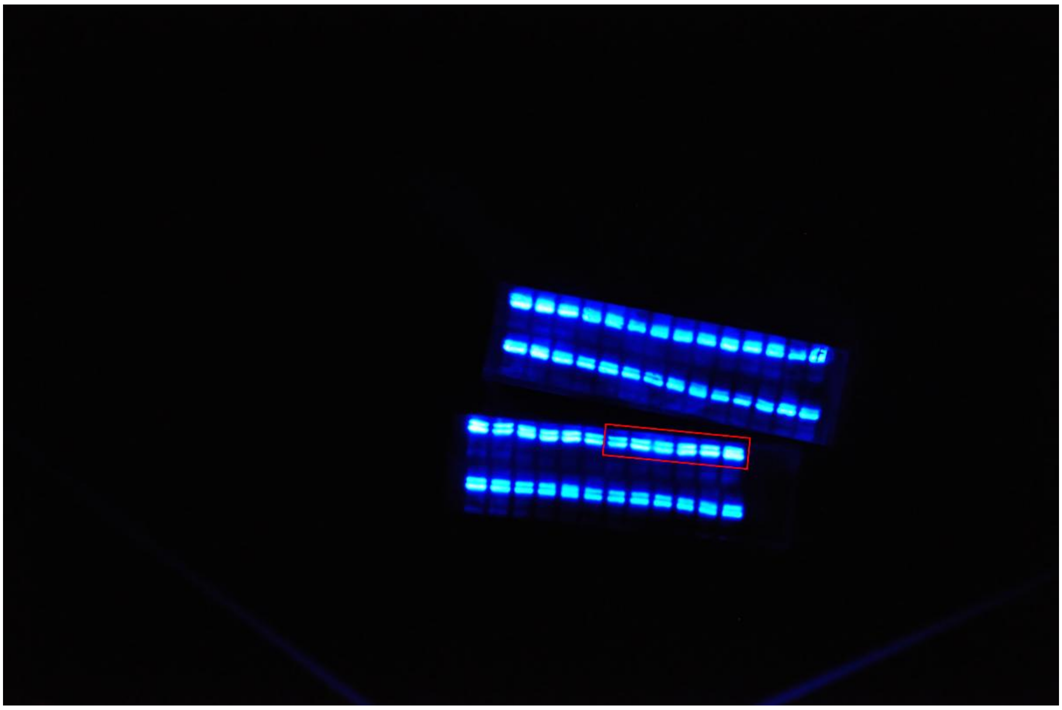

Figure S12. In the red box, from left to right, the total ERK1/2 of blank (untransfected) cells, control (transfected with empty vector pEGFP-N1) cells, and AjGPER1-EGFP expressing cells treated with DMSO or BPA (100 nM) for 10 min, respectively. Corresponding to Figure 7A in the manuscript.

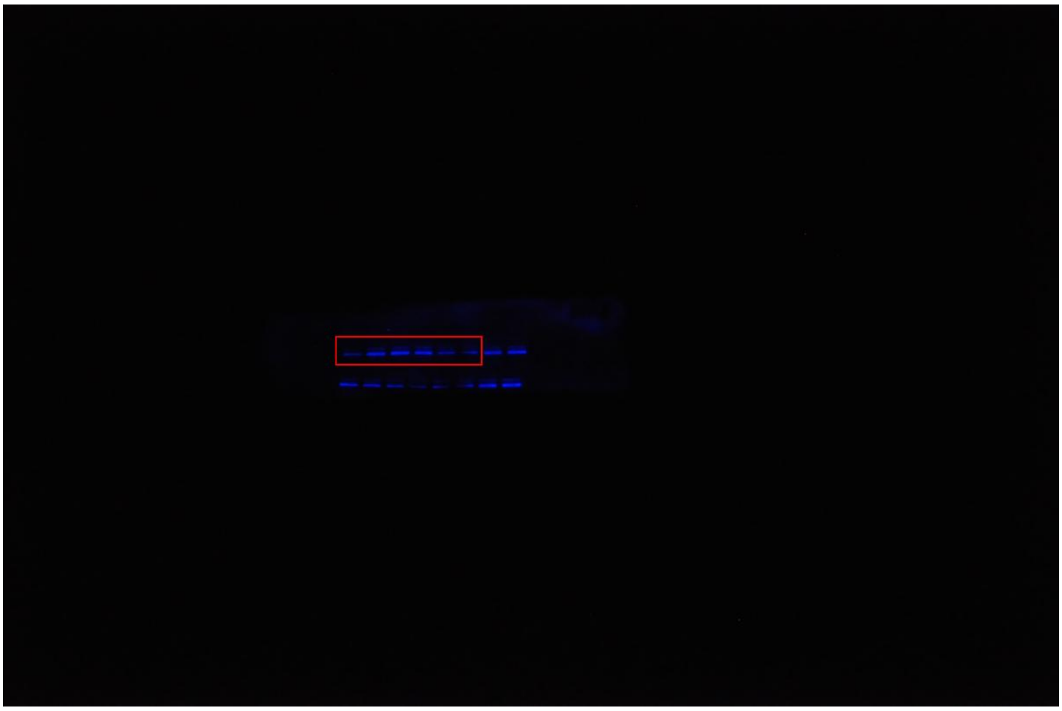

Figure S13. In the red box, from left to right, time course of BPA (100 nM) treatment induced ERK1/2 phosphorylation in AjGPER1-EGFP-expressing cells. Corresponding to Figure 7B in the manuscript.

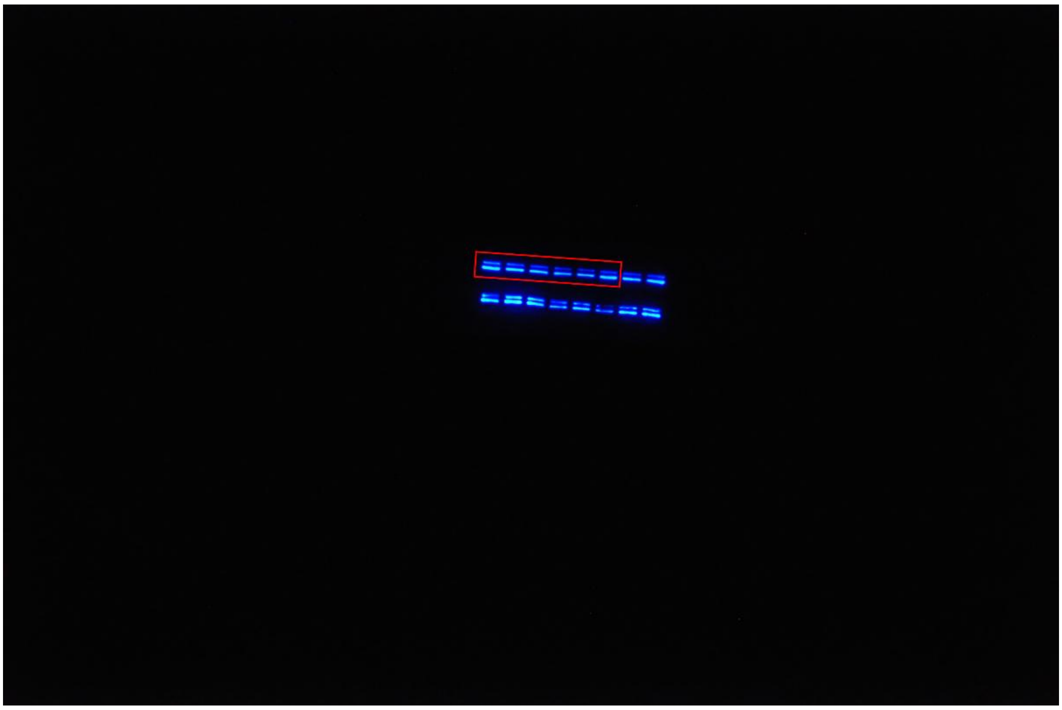

Figure S14. In the red box, from left to right, time course of BPA (100 nM) treatment total ERK1/2 in AjGPER1-EGFP-expressing cells. Corresponding to Figure 7B in the manuscript.

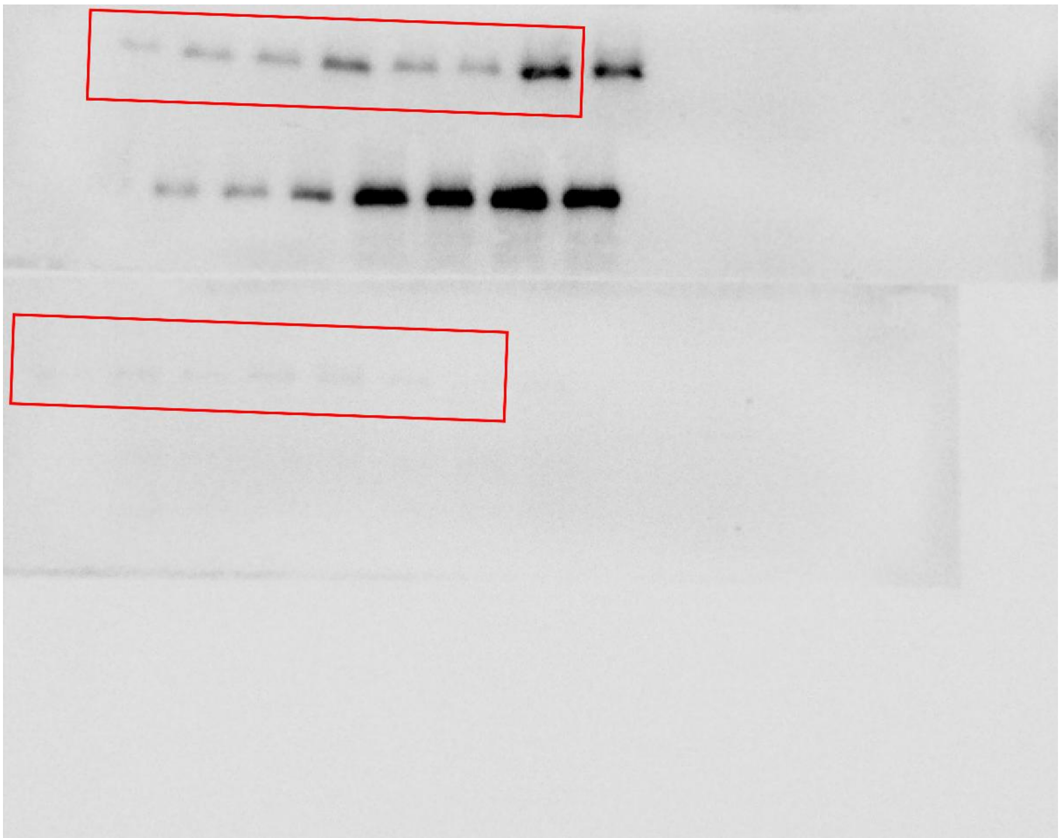

Figure S15. In the red box, from top to bottom, from left to right, the phosphorylated ERK1/2 in *A. japonicus* ovary and respiratory tree tissues actived by E_2 and BPA. Corresponding to Figure 8B in the manuscript.

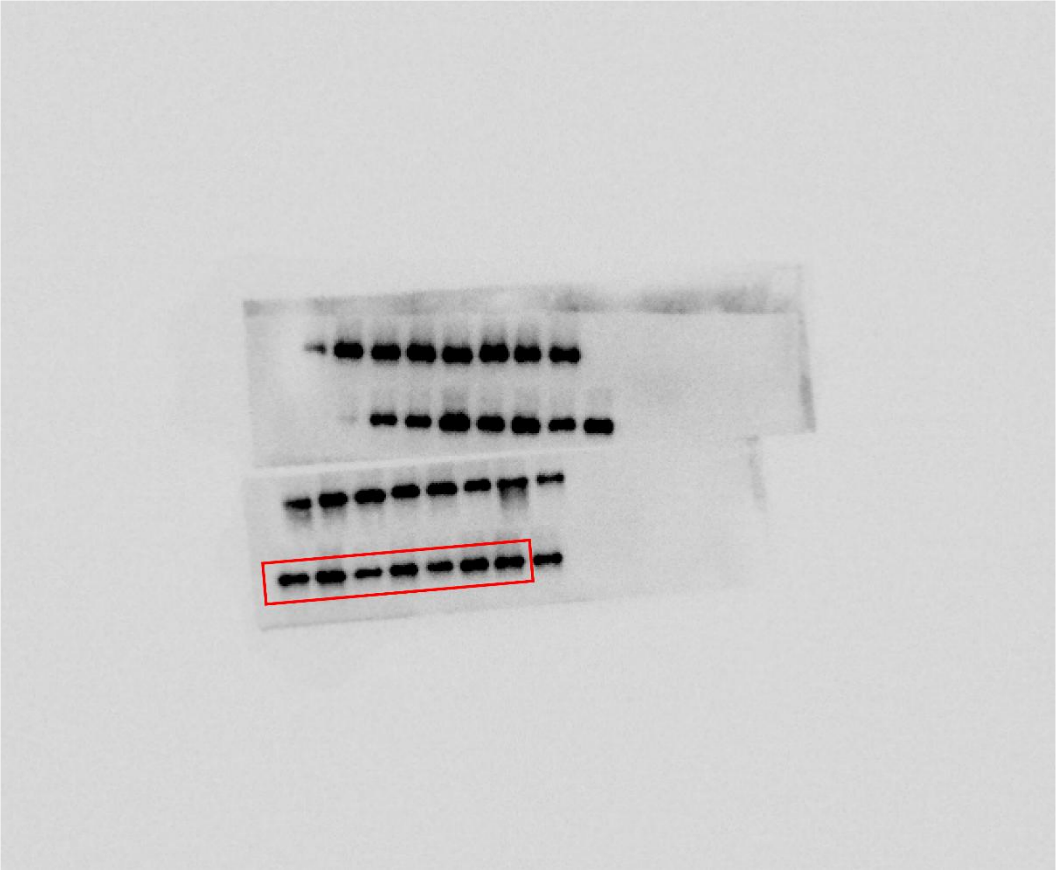

Figure S16. In the red box, from left to right, the total ERK1/2 in *A. japonicus* respiratory tree tissues. Corresponding to Figure 8B in the manuscript.

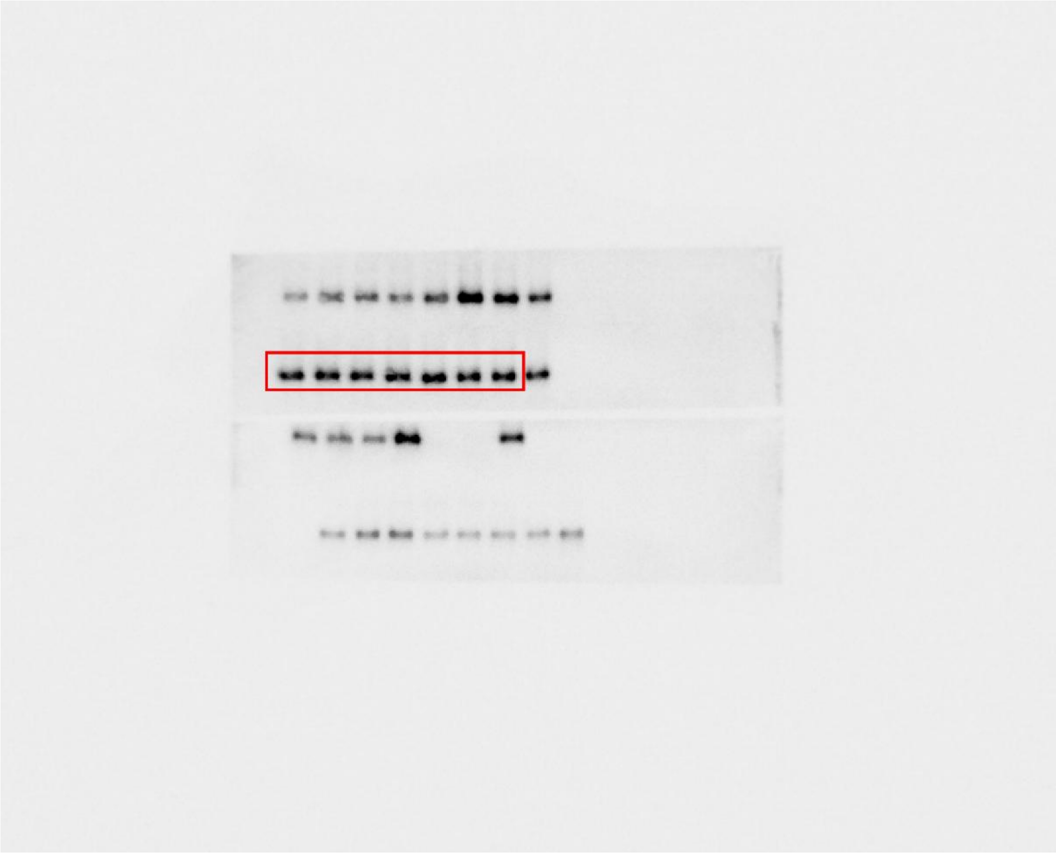

Figure S17. In the red box, from left to right, the total ERK1/2 in *A. japonicus* ovary. Corresponding to Figure 8B in the manuscript.
