# Supplementary material for: Bisphenol A (BPA) Directly Activates the G Protein-Coupled Estrogen Receptor 1 and Triggers the Metabolic Disruption in the Gonadal Tissue of Apostichopus japonicus
Source: Biology (Basel). 2023 May 31;12(6):798. doi: 10.3390/biology12060798 (PMC10295501; doi:10.3390/biology12060798)
Supplement: Supplementary file 1 [file biology-12-00798-s001.zip › Tables S1-S3 & Figure S1.pdf]

Bisphenol A (BPA) directly activates the G protein-coupled estrogen receptor 1 and triggers the metabolic disruption in the gonadal tissue of *Apostichopus japonicus*

Jieyi Yuan[1,†], Jingwen Yang[1,†], Xiuwen Xu[1], Zexianghua Wang[1], Zhijing Jiang[1], Zhiqing Ye[1], Yucheng Ren[1], Qing Wang[2,*], Tianming Wang[1,*]

* Correspondence: Tianming Wang: wangtianming@zjou.edu.cn; Qing Wang: qingwang@yic.ac.cn

Table S1. GenBank accession numbers corresponding to the sequence used for phylogenetic tree analysis and multiple sequence alignment.

Gene type	abbreviation	GenBank accession numbers
Estrogen receptor α	HsERα	NP_000116.2
	MmERα	NP_001289460
	CfERα	XP_533454
	SsERα	NP_999385.1
	GgERα	NP_990514.1
	DrERα	NP_694491.1
	CaERα	XP_026089164.1
	XlERα	NP_001083085
	BmERα	XP_014332422
	LcoERα	NP_001290305
	LcfERα	XP_018528117
	HcERα	XP_019750588
	CsERα	XP_008310919
	OsERα	XP_029644783
	LaERα	XP_013404576
	OgERα	XP_046148097
	MaERα	XP_020443618
	CmERα	XP_007892594
Estrogen receptor β	HsERβ	NP_001428
	MmERβ	NP_034287
	CfERβ	XP_038445068
	SsERβ	NP_001001533
	GgERβ	NP_990125
	DrERβ	NP_777287
	CaERβ	XP_026079489
	XlERβ	NP_001124426
	BmERβ	XP_005898314
	LcmERβ	XP_005986456
	LcoERβ	XP_010737659
	LcfERβ	XP_018538032
	HcERβ	XP_019726315
	CsERβ	NP_001281164
	OgERβ	XP_046147746
	MaERβ	XP_020459576
	RtERβ	XP_020380159
G protein-coupled estrogen receptor 1	HsGPER1	NP_001035055
	MmGPER1	NP_084047

	CfGPER1	XP_005621261
	SsGPER1	XP_003124292
	GgGPER1	NP_001155877
	DrGPER1	NP_001122195
	CaGPER1	XP_026056231
	XlGPER1	XP_018091862
	BmGPER1	XP_014338621
	LcmGPER1	XP_006010235
	LcoGPER1/LcGPER1	XP_010728847
	LcfGPER1	XP_018537294
	HcGPER1	XP_019731372
	CsGPER1	XP_008312438
	OgGPER1	XP_046163069
	MaGPER1	XP_020466193
	CmGPER1	XP_007901691
Kisspeptin1 receptor	HsKISS1R	NP_115940
	MmKISS1R	NP_444474
	SsKISS1R	NP_001038089
	DrKISS1R	NP_001099149
	CaKISS1R	XP_026051331
	XlKISS1R	XP_018080755
	LcoKISS1R	XP_010727548
	LcfKISS1R	XP_018528723
	HcKISS1R	XP_019739218
	CsKISS1R	XP_008332638
	OgKISS1R	XP_046178411
	CmKISS1R	XP_007898035

Table S2. The primers used in the experiments

Primer	Sequence
AjGPER1-F	CTTCAACACAGTTCTTCTGTCAAGA
AjGPER1-R	TGTCGACCTGTATAATCTCGTCAT
q-AjGPER1-F	GTCGTAGCGACTGTCCATATAGTT
q-AjGPER1-R	CATCTTGCCTGTTCTGATACTGACT
β-tublin-F	CACCACGTGGACTCAAAATG
β-tublin-R	GAAAGCCTTACGACGGAACA

Table S3. Multiple sequence alignment

abbreviation	% identity	alignment length	E value
AjGPER1	100.000	398	0.0
HsGPER1	27.891	294	$1.59e^{-26}$
GgGPER1	26.074	326	$5.48e^{-24}$
MmGPER1	25.965	285	$1.59e^{-23}$
DrGPER1	28.045	353	$2.20e^{-23}$
XlGPER1	28.472	288	$1.31e^{-22}$
HcGPER1	27.445	317	$1.94e^{-20}$
MaGPER1	28.615	325	$3.99e^{-20}$
LcGPER1	27.066	351	$6.07e^{-20}$
CmGPER1	25.402	311	$6.26e^{-19}$

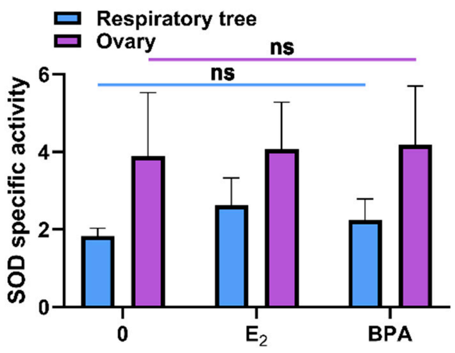

Figure S1. Effects of 4 h exposure to E_2 or BPA on SOD enzyme activity in the respiratory tree and ovary tissue of *A. japonicus*. Values shown are multiples of the lowest activity of the control group expressed as means ± SEM (n = 3). The bars of different colors represent different tissues. Data were analyzed by using one-way ANOVA followed by Tukey's multiple comparison tests (ns $P > 0.05$).
